# Efficacy of Procaine, With and Without Epinephrine, Compared to Lidocaine in Local Anesthesia for Calves Before Thermocautery Disbudding

**DOI:** 10.1111/jvp.13493

**Published:** 2024-11-27

**Authors:** Magdy Adam, Annemari Jokela, Kati Salla, Riikka Aho, Marja Raekallio, Laura Hänninen, Ann‐Helena Hokkanen

**Affiliations:** ^1^ Department of Production Animal Medicine, Faculty of Veterinary Medicine University of Helsinki Helsinki Finland; ^2^ Research Centre for Animal Welfare, Faculty of Veterinary Medicine University of Helsinki Helsinki Finland; ^3^ Department of Pharmacology, Faculty of Veterinary Medicine Beni‐Suef University Beni Suef Egypt; ^4^ Department of Equine and Small Animal Medicine, Faculty of Veterinary Medicine University of Helsinki Helsinki Finland

**Keywords:** calves, disbudding, lidocaine, pain, procaine

## Abstract

Within the European Union, the use of lidocaine in food‐producing animals is restricted due to concerns over human safety. This study compared the clinical effectiveness of procaine, with and without epinephrine, against lidocaine in pain alleviation during thermocautery disbudding in xylazine‐sedated calves. The efficacy of local blocks was assessed through needle pricks, and the behavioral reactions to disbudding were scored. Post‐disbudding pain was subjectively evaluated, and pressure pain threshold and tactile sensitivity around the horn bud were assessed at intervals. Blood was collected at intervals for plasma cortisol analysis. No significant differences were found between the groups in the needle prick test (*p* = 0.329) and the disbudding score (*p* = 0.855). Pain scores and quantitative sensory tests showed no significant differences between the lidocaine and procaine‐epinephrine groups. Conversely, tactile sensitivity and pain scores were significantly higher, and pressure pain thresholds were significantly lower with procaine alone than in other groups. Elevated cortisol concentrations were observed in all groups before disbudding compared to the baselines. The results suggest that procaine combined with epinephrine appears to be a safe and effective alternative to lidocaine for calf disbudding. Cortisol concentrations as an indicator of pain in xylazine‐sedated calves appear inadequate.

## 1 Introduction

Disbudding, the removal or destruction of horn buds, is practiced globally in young dairy calves. This procedure is carried out for several reasons, including facilitating easy handling and transportation, reducing the likelihood of carcass wastage due to bruising, and enhancing safety within the herd (Cozzi et al. 2015). Nevertheless, disbudding induces both severe acute pain (Winder et al. 2018) and long‐lasting postoperative pain (Adcock et al. 2023), leading to notable behavioral and physiological responses. This makes disbudding a significant welfare concern (Herskin and Nielsen 2018). Thus, recognizing the need for pain alleviation, many European countries, including Finland, require adequate sedation and analgesia during disbudding, aligning with the (Council Directive 2008/119/EC).

Local anesthesia, particularly through cornual nerve blocks, plays a pivotal role in alleviating pain during disbudding in calves. A successful cornual nerve block not only mitigates the immediate pain response during disbudding but also delays the onset of post‐procedural pain, especially when combined with nonsteroidal anti‐inflammatory drugs (Winder et al. 2018). Lidocaine is the most widely used and extensively studied local anesthetic in disbudding and dehorning studies (Herskin and Nielsen 2018; Winder et al. 2018). Clinically, local anesthesia with lidocaine attenuated behaviors associated with the immediate pain response (e.g., tail wagging, head movements, tripping, and rearing) and those indicative of postoperative pain (head rubbing, head shaking, and ear flicking) (Heinrich et al. 2010; Huber et al. 2013; Mintline et al. 2013; Herskin and Nielsen 2018; Adcock, Cruz, and Tucker 2020). Furthermore, lidocaine has been shown to dampen the initial rise in plasma cortisol, indicative of stress, during the respective duration of action following disbudding (Herskin and Nielsen 2018; Winder et al. 2018). Additionally, lidocaine administration improved the growth rate in medicated calves compared to those receiving no pain relief for disbudding (Bates et al. 2016; Cuttance et al. 2019).

However, lidocaine poses a risk to consumers when used in the food‐producing animals due to potential exposure to one of its metabolites, 2,6‐xylidine, which has been shown to have genotoxic characteristics in vivo (European Medicines Agency 2015). Consequently, procaine is currently the only approved local anesthetic agent for cattle within the European Union. Thus, lidocaine can be used only under the so‐called cascade rule (off‐label/extralabel use) if the use of procaine is contraindicated (Council Regulation EU 2019/6). However, the effectiveness of procaine when used in a local nerve block before the thermocautery disbudding in calves remains largely unknown, with only limited studies available on this topic. For instance, in one study, calves were disbudded 20 min after procaine infiltration, but the efficacy of local anesthesia was not reported (Huber et al. 2013). More recently, we reported that only one calf of 27 animals exhibited a slight reaction to disbudding when sedated with intramuscular (im) xylazine combined with local infiltration of procaine (4.5 mg kg^−1^) 15 min before thermocautery disbudding (Adam et al. 2021). By contrast, Thomsen, Hansen, and Herskin (2021) found that approximately 40% of calves showed signs of inadequate local anesthesia, such as kicking or head lifting, while sedated with xylazine (0.7 mg kg^−1^ im) and given cornual nerve block with procaine before thermocautery disbudding. Thus, concerns persist regarding the adequacy of procaine in alleviating disbudding pain (Nielsen et al. 2023). However, a new product that combines procaine and epinephrine has been recently authorized for veterinary use in Europe (European Medicines Agency 2019) and may offer better pain alleviation. Unfortunately, it has not yet been evaluated in calves. Therefore, additional research is crucial to further understand the suitability and effectiveness of procaine, especially when combined with epinephrine, for pain alleviation in association with disbudding.

Furthermore, from a literature search, a comparative assessment of lidocaine and procaine (with and without epinephrine) in terms of efficacy in calves during thermocautery disbudding has yet to be conducted. Therefore, the study objective was to evaluate the efficacy of these three local anesthetic products during disbudding and to assess their effects on post‐disbudding pain in calves. It was hypothesized that these products, when used for cornual nerve blocks, would provide a sufficient level of local anesthesia for disbudding. Additionally, it was hypothesized that the efficacy of procaine combined with epinephrine would be comparable to that of lidocaine.

## 2 Materials and Methods

### 2.1 Animals, Housing, and Feeding

A total of 36 Ayrshire and Blonde D'Aquitaine calves, 31 males and 5 females, aged 23.6 ± 4.9 days (mean ± SD) and body mass of 75 ± 11.8 kg were enrolled in the study at the Viikki Teaching and Research Facility at the University of Helsinki, Finland. Calves were accommodated in straw‐bedded single boxes during their first week of life and received milk from teat buckets. Permanent visual contact among the calves was ensured. Subsequently, they were moved to sawdust‐bedded pens, with milk available through an automatic feeder and free access to grass silage, hay, and water. Inclusion criteria comprised normal birth weight, an unremarkable clinical examination, and normal blood hematology and biochemistry analyses. Calves showing signs of disease or having an infectious disease within 2 weeks before disbudding were excluded. The study received approval from the National Animal Experiment Board (permission number ESAVI/9272/2018), meeting European Union regulations and ARRIVE guidelines. To ensure replicability, these guidelines were followed for comprehensive reporting, not solely for study approval. Approval was also granted by the Finnish Medicines Agency.

### 2.2 Study Preparations and Sedation

One day before disbudding, the calves underwent a thorough clinical examination, had the hair around the horn buds and overlying the jugular veins clipped, and were weighed using a digital scale. Subsequently, a blood sample was collected for hematology and biochemistry analyses. On the day of disbudding, animals were sedated 30 min before disbudding (−30′) with xylazine im (0.2 mg kg^−1^, Nefrasin vet 20 mg mL^−1^, Le Vet B.V., Netherlands). Upon achieving a suitable level of sedation with recumbency, and no response to handling, a short‐term venous catheter (16‐gauge × 60 mm, Intraflon 2 IV cannula, Vygon, France) was aseptically inserted into one jugular vein and used for the subsequent blood samplings.

### 2.3 Treatment Groups

**FIGURE 1:**
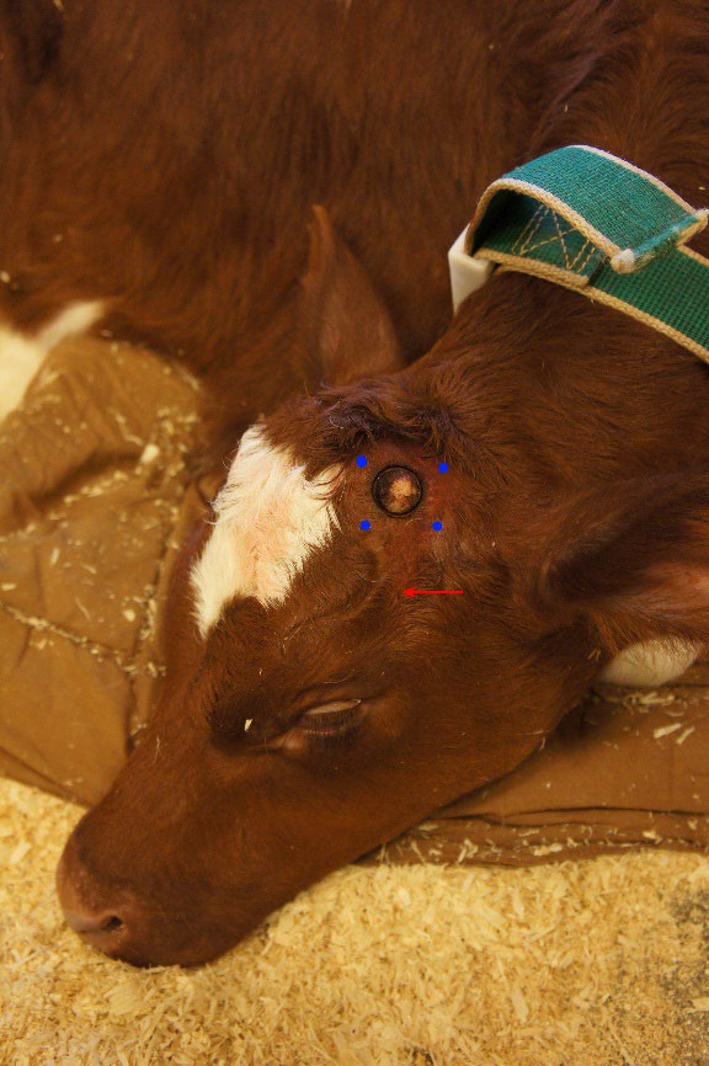
A photograph of a calf's head shows the four spots (marked with blue dots) selected around each horn bud to measure pressure pain threshold with algometry and tactile pain sensitivity with von Frey monofilaments. The red arrow indicates the location of the needle placement for desensitizing the cornual nerve.

Calves were randomly assigned to three equal groups (www.randomization.com). Fifteen minutes after sedation (−15′), bilateral cornual nerve blocks were performed as previously described (Fierheller et al. 2012). Injectate was deposited halfway between the horn bud base and the lateral canthus of the eye (Figure 1). The three treatment groups were: (1) Procaine; procaine hydrochloride (4.5 mg kg^−1^, Procamidor vet 20 mg mL^−1^, Richter pharma AG, Austria) (2) Procaine‐epi; procaine and epinephrine (2.25 mg kg^−1^, Procamidor Comp vet 40 mg mL^−1^ [procaine] and 0.036 mg mL^−1^ [epinephrine], Richter pharma AG, Austria), or (3) Lidocaine; lidocaine hydrochloride (3 mg kg^−1^ Lidor vet 20 mg mL^−1^, Richter pharma AG, Austria). Calculated drug volumes were divided equally between two cornual nerve blocks. At the time of performing the nerve blocks, meloxicam (0.5 mg kg^−1^, Metacam, 20 mg mL^−1^, Boehringer Ingelheim, Germany) was given subcutaneously (SC). The efficacy of local anesthesia was assessed 5 min after local blocks were completed (−10′) and evaluated by the reaction to a fresh 1.5‐inch, 23‐gauge, sterile needle pricked over and around the horn buds (Bates et al. 2019). If there was a reaction, the test was repeated after an additional 5 min (−5′) had elapsed and, if there was still a reaction, a third series of needle pricks was performed immediately before disbudding (0′) and the calf would have been excluded. The same investigator, blinded to the treatments, assessed pain and measured local variables.

### 2.4 Disbudding

Thirty minutes after sedation (0′), a preheated (approximately 650°C) portable butane gas cautery‐iron dehorner (Express Gas Portable Dehorner with a 20 mm head, Albert Kerbl GmbH, Germany) was applied over each horn bud for 5–10 s depending on the bud size. The reaction during disbudding was scored for individual horn bud with a 4‐point scale, modified from Bates et al. (2019), where 0 = no response, 1 = slight movement of the body, tail wagging, ear flick, 2 = mild struggling with limbs, and 3 = massive struggling involving the whole body.

### 2.5 Pain, Tactile Sensitivity, and Pressure Pain Threshold Assessments

Pain severity was scored at baseline, 60, 90, 120, 150, 180, 240, 360 min, and 24 h after disbudding using a multidimensional pain score (0–21; where 0 is no pain and 21 the worst possible pain), modified from Mirra et al. (2018) (Appendix A), alongside a 100 mm visual analog scale (VAS), where 0 mm is no pain and 100 mm is the worst possible pain.

Tactile sensitivity was assessed at baseline, 90, 120, 150, 180, 240, 360 min, and 24 h after disbudding with a set of 17 von Frey monofilaments (Aesthesio, Ugo Basile, Italy) ranging from 4.39 to 193 g mm^−2^ of force, as previously described by Mintline et al. (2013) and Mirra et al. (2018). Briefly, animals were gently restrained, and then, starting with the thinnest monofilament, four points around each bud were touched (Figure 1). If there was no response, the next monofilament was applied until a behavioral response was observed, and then the assessment at that point was ceased. Responses were scored as presented in Appendix B.

The pressure pain thresholds were evaluated at baseline, 90, 120, 150, 180, 240, 360 min, and 24 h after disbudding with a pressure algometer (FPN 200–Algometer, Wagner Instruments, USA). Pressure at 2 N s^−1^ rate was applied to the same four points used for von Frey until the animal exhibited signs of discomfort or the applied force reached 70 N, whereas 10 N was used as a lower cutoff when the animal showed a reaction to the probe before any further pressure was applied. Peri‐bud swelling was assessed at baseline, 240 and 360 min, and 24 h after disbudding with a 3‐point scale (0 is no swelling, while 1 and 2 are mild and severe swelling, respectively).

### 2.6 Blood Samples

Four milliliters of blood was collected into lithium heparin tubes at baseline, immediately before disbudding (0′), and 30, 60, 90, 120, 150, 180, 240, 360 min, and 24 h after disbudding. Samples were kept in iced water, centrifuged within 30 min, and plasma was frozen at −20°C until analysis. Cortisol was assayed in duplicate and quantified by radioimmunoassay using a commercial kit (ImmuChem Cortisol Coated Tube 125I RIA kit, MP Biomedicals, Diagnostics Division, Orangeburg, NY, USA).

### 2.7 Statistical Analysis

The sample size estimate was derived from prior studies (Mirra et al. 2018; Casoni et al. 2019), where ten calves per group would be needed to detect a 10 ± 8 N difference in pressure pain threshold at the time when local anesthetic effect is wearing off and provide a power of 80% with an alpha level of 0.05. Within each calf, disbudding score and pressure pain thresholds were averaged to calculate one value at each time point for both horn buds, which was used for further statistical analysis. For tactile sensitivity, the four scores obtained for each horn were summed to obtain a total score, and then were averaged to calculate one value per each calf at each time point for use in statistical analysis. The Shapiro–Wilk tests demonstrated that the distributions of the variables were non‐normal; consequently, non‐parametric analyses were utilized. Non‐parametric tests are also well‐suited for ordinal data, as they can analyze ranked data without assuming equal intervals between categories, making them ideal for this study's categorical scoring approach. The Kruskal–Wallis test was employed to compare variables (namely, pain and disbudding scores, pressure pain thresholds, tactile sensitivity, and cortisol concentrations) between groups at the same time point. When significant differences were found, multiple Mann–Whitney *U* tests were conducted, with *p* values adjusted for multiple tests with a Bonferroni correction. The pairwise comparisons to the baseline within each group were performed with the Wilcoxon test where applicable. Chi‐squared tests were used to determine differences in the number of calves reacting to the needle prick. Additionally, Spearman's rank correlations were performed to assess the associations between plasma cortisol concentrations and pain scores. The level of significance was set at 0.05. All analyses were performed using commercial software (IBM SPSS Statistics for Windows Version 29, IBM Corp., NY, USA).

## 3 Results

All local anesthetic products effectively produced a cornual nerve block sufficient for disbudding, with no significant differences in the needle prick test among the groups. Ten minutes before disbudding (−10′), there were no reactions to the needle prick in the Procaine group, whereas with Procaine‐epi and Lidocaine, only one animal in each group exhibited a reaction (*p* = 0.329), and at −5′, no reactions were observed in any of the groups. Likewise, no significant differences in disbudding scores were noted between the groups nor in peri‐bud swelling scores (Table 1).

**TABLE 1:** Median (range) of disbudding score and peri‐bud swelling score over both horn buds in 36 calves sedated with xylazine at baseline (time −30′) and receiving cornual nerve blocks with either procaine alone (Procaine), procaine combined with epinephrine (Procaine‐epi), or lidocaine (Lidocaine) 15 min prior to thermocautery disbudding (time 0′).

	Time	Group			
		Procaine	Procaine‐epi	Lidocaine	*p*
Disbudding score (0–3)		0 (0–1)	0 (0–0.5)	0 (0–1.5)	0.855
Peri‐bud swelling score (0–2)	BL	0 (0–0)	0 (0–0)	0 (0–0)	1.00
	240′	1 (1–2)	1 (0–1)	1 (0–1)	0.694
	360′	1 (1–2)	1 (1–2)	1 (1–2)	0.502
	24 h	1 (1–2)	1 (1–2)	1 (1–1)	0.598

*Note:* BL, baseline; 240′ and 360′, 240 and 360 min after disbudding; and 24 h, 24 h after disbudding.

**FIGURE 2:**
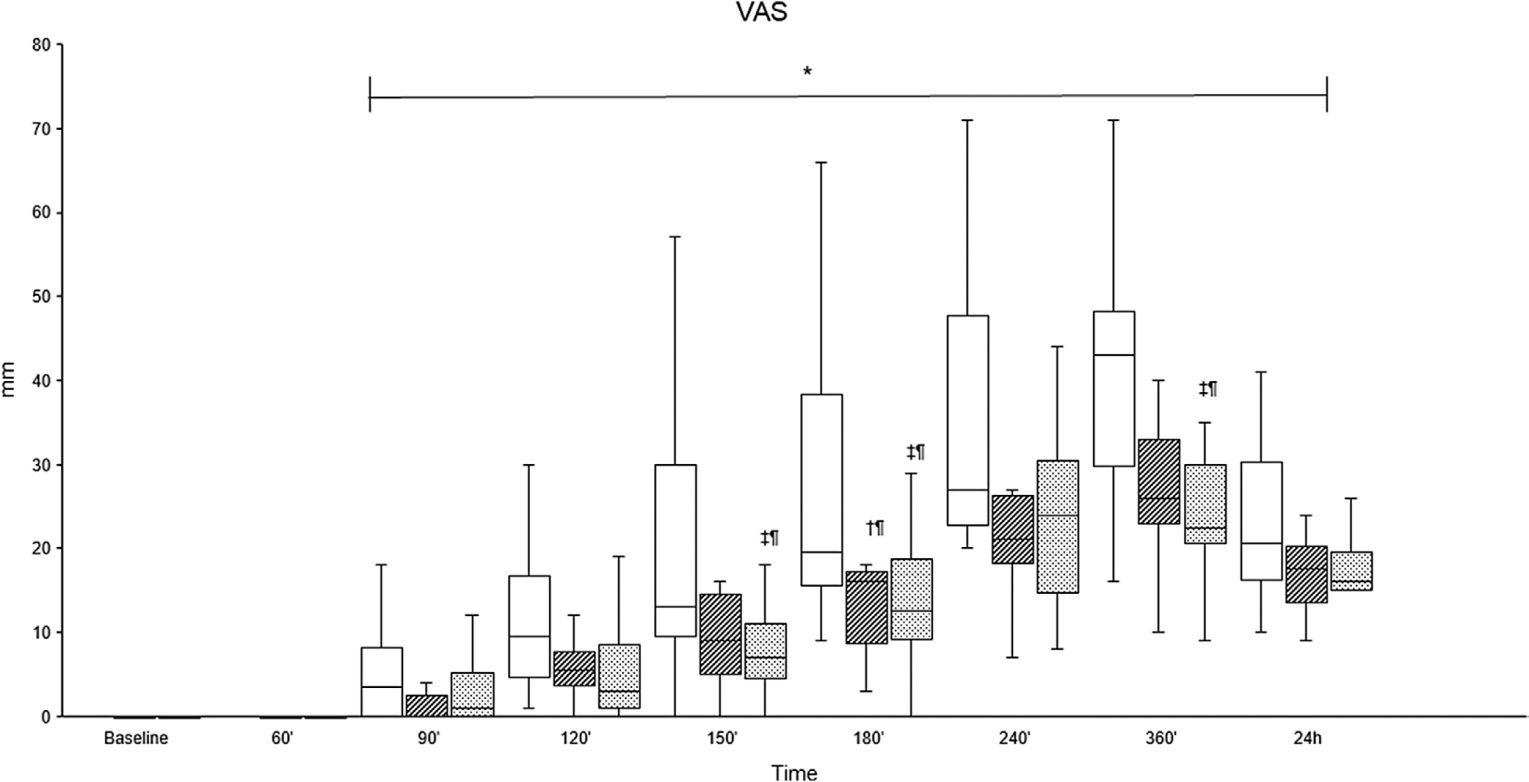
Median (interquartile range) of visual analog pain score (VAS) in 36 calves sedated with xylazine at baseline (time −30′) and receiving cornual nerve blocks with either procaine alone (Procaine, empty), procaine combined with epinephrine (Procaine‐epi, diagonal lines), or lidocaine (Lidocaine, dots) 15 min prior to thermocautery disbudding (time 0′). *Significant difference from baseline (BL) within each group (*p* < 0.05). ^†^Significant difference between Procaine and Procaine‐epi, and ^‡^between Procaine and Lidocaine; significance levels are indicated as follows: ^*¶*^ *p* < 0.05, ^#^ *p* < 0.01, and ^††^ *p* < 0.001.

**FIGURE 3:**
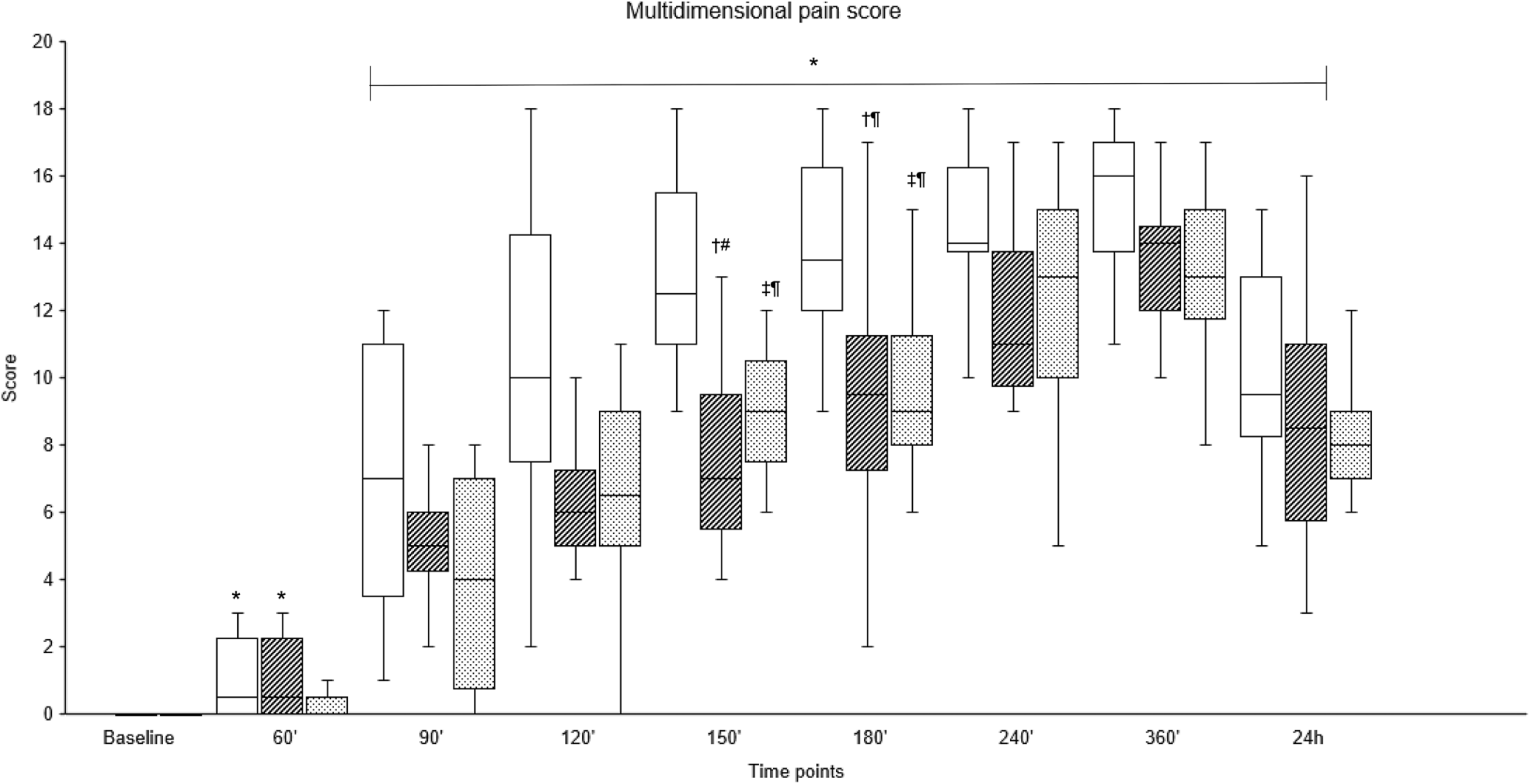
Median (interquartile range) of multidimensional pain score in 36 calves sedated with xylazine at baseline (time −30′) and receiving cornual nerve blocks with either procaine alone (Procaine, empty), procaine combined with epinephrine (Procaine‐epi, diagonal lines), or lidocaine (Lidocaine, dots) 15 min prior to thermocautery disbudding (time 0′). *Significant difference from baseline (BL) within each group (*p* < 0.05). ^†^Significant between Procaine and Procaine‐epi, and ^‡^between Procaine and Lidocaine; significance levels are indicated as follows: ^*¶*^ *p* < 0.05, ^#^ *p* < 0.01, and ^††^ *p* < 0.001.

**FIGURE 4:**
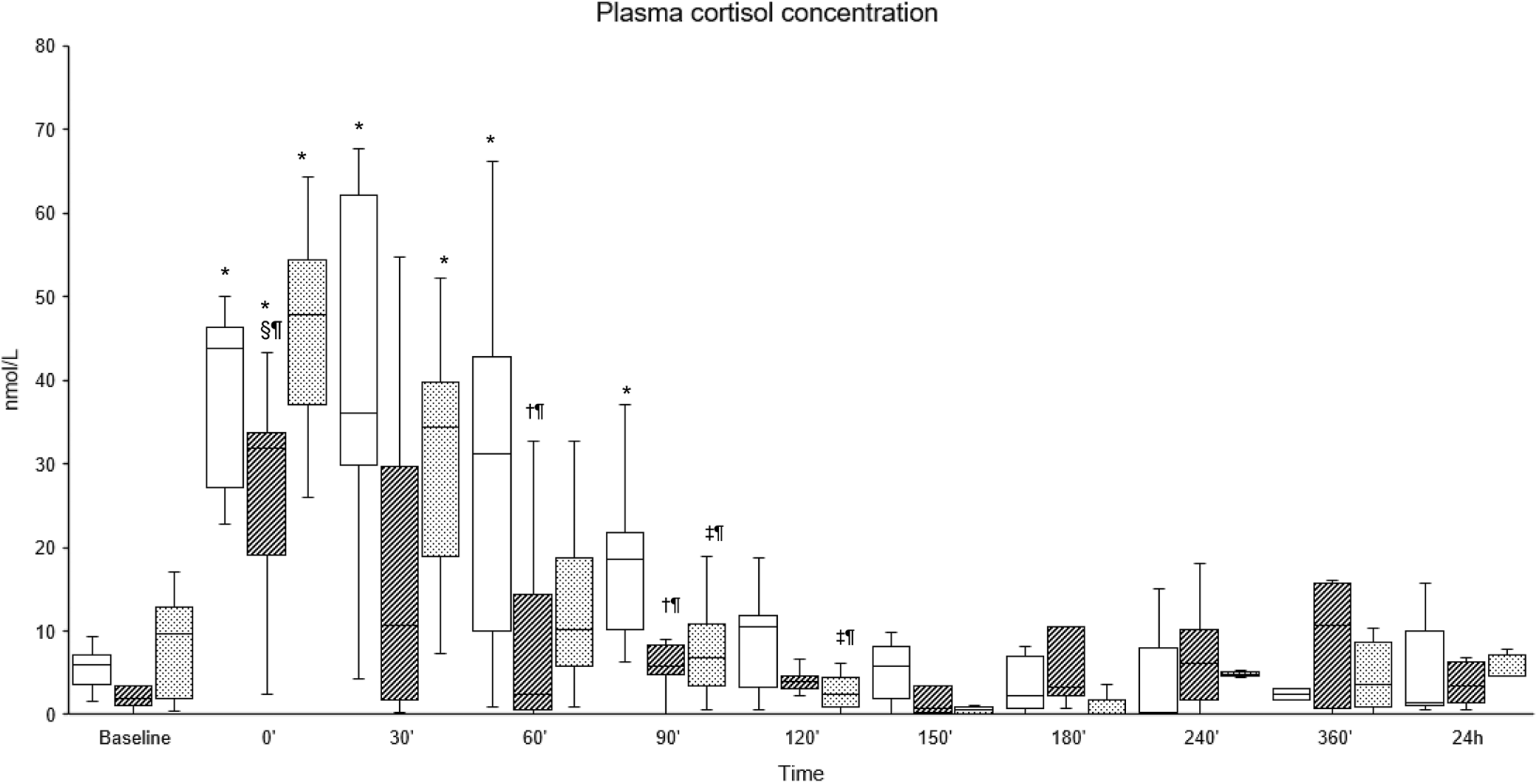
Median (interquartile range) of plasma cortisol concentrations in 36 calves sedated with xylazine at baseline (time −30′) and receiving cornual nerve blocks with either procaine alone (Procaine, empty), procaine combined with epinephrine (Procaine‐epi, diagonal lines), or lidocaine (Lidocaine, dots) 15 min prior to thermocautery disbudding (time 0′). *Significant difference from baseline (BL) within each group (*p* < 0.05). ^†^Significant between Procaine and Procaine‐epi, and ^‡^between Procaine and Lidocaine; significance levels are indicated as follows: ^*¶*^ *p* < 0.05, ^#^ *p* < 0.01, and ^††^ *p* < 0.001.

Pain scores were significantly higher between 150′ and 360′ in the Procaine group compared to the Procaine‐epi and Lidocaine groups (Figures 2 and 3). The tactile sensitivity mean sum score of both horn buds was significantly higher in the Procaine group compared to the Procaine‐epi and Lidocaine groups between 90′ and 180′ (Table 2). At the same time, the mean pressure pain thresholds of both horn buds were significantly lower during the same time frame (Table 2). Plasma cortisol concentrations were significantly higher immediately before the disbudding (0′) in all treatment groups compared to their baselines (Figure 4). In addition, plasma cortisol concentrations were weakly, but significantly, correlated to pain scores with correlation coefficients of −0.192 and −0.256 to VAS and multidimensional pain scores, respectively.

**TABLE 2:** Median (range) of pressure pain thresholds with algometry and tactile sensitivity with von Frey monofilaments over both horn buds in 36 calves sedated with xylazine at baseline (time −30′) and receiving cornual nerve blocks with either procaine alone (Procaine), procaine combined with epinephrine (Procaine‐epi), or lidocaine (Lidocaine) 15 min prior to thermocautery disbudding (time 0′).

Variable	Group	Time							
		BL	90′	120′	150′	180′	240′	360′	24 h
Pressure pain threshold	Procaine	46 (23–70)	59 (11–70)	27 (10–67)	16 (10–47)*	13 (10–24)*	10 (10–27)*	11 (10–18)*	13 (10–28)*
	Procaine‐epi	56 (27–70)	70 (63–70)* ^†#^	70 (25–70))* ^†††^	66 (11–70)^†*¶*^	39 (10–66)	25 (10–57)^*†*¶*^	14 (10–31)*	13 (10–40)*
	Lidocaine	63 (28–70)	70 (58–70))* ^‡*¶*^	70 (39–70)^‡††^	67 (13–70)^‡#^	38 (12–70)^‡#^	13 (10–48)*	15 (10–36)*	15 (10–27)*
Tactile sensitivity	Procaine	6 (0–16)	8 (0–24)	15 (1–24)*	20 (6–24)*	22 (12–24)*	24 (20–24)*	24 (22–24)*	23 (20–24)*
	Procaine‐epi	3 (0–12)	0 (0–6))* ^†#^	0 (0–16) ^†*††*^	2 (0–24)^†#^	5 (0–24)^†#^	20 (1–24)*	22 (11–24)*	22 (5–24)*
	Lidocaine	3 (0–18)	0 (0–8)^‡*¶*^	1 (0–24)^‡*#*^	4 (0–23)^‡*¶*^	10 (0–24)*	24 (8–24)*	24 (12–24)*	24 (10–24)*

*Note:* ^†^Significant difference between Procaine and Procaine‐epi, and ^‡^between Procaine and Lidocaine; significance levels are indicated as follows: ^*¶*^ *p* < 0.05, ^#^ *p* < 0.01, and ^††^ *p* < 0.001.

* Significant difference from baseline (BL) within each group (*p* < 0.05).

## 4 Discussion

To the authors' knowledge, this study represents the first attempt to compare the efficacy of procaine, with and without epinephrine, to the commonly used local anesthetic lidocaine for cornual nerve block in calves before thermocautery disbudding. The findings demonstrate that all three local analgesic products provided sufficient anesthesia for disbudding, with no significant differences observed in the needle prick test and reaction to disbudding. Similarly, no significant differences were noted in any tested variables between the Procaine‐epi and Lidocaine groups throughout the observation period. Conversely, pain scores and tactile sensitivity were significantly higher, while pressure pain thresholds were significantly lower with Procaine compared to Procaine‐epi and Lidocaine.

In the current study, the efficacy of procaine as a local anesthetic for the disbudding of calves aligns with previous research when it was used for local anesthesia and combined with xylazine sedation prior to thermocautery disbudding (Adam et al. 2021). In contrast, Thomsen, Hansen, and Herskin (2021) reported that 42% of disbudded calves showed signs of inadequate local anesthesia, such as kicking or head lifts. Several factors may contribute to this discrepancy: First, variations in the time interval from administration of local anesthesia to disbudding (ranging from 2 to 35 min, with a mean of 16 min) in the Thomsen, Hansen, and Herskin (2021) study could have led to some calves being disbudded before procaine's effects entirely took hold. The onset of action of local anesthetics is influenced by their lipid solubility, with high solubility indicating greater potency and faster onset (Day and Skarda 1991). Moreover, the greater lipid solubility of local anesthetics not only enhances potency but also enables more rapid diffusion through nerve sheaths and neural membranes (Becker and Reed 2012). Procaine is characterized by low lipid solubility with a partition coefficient of 0.02 and pKa of 8.9 (Day and Skarda 1991), which diminishes its ability to penetrate tissues and prolongs the onset of action (Day and Skarda 1991; Tetzlaff 2000). Thus, manufacturer guidelines recommend a waiting period of 5–10 min between drug administration and disbudding (Richter Pharma 2017, Procamidor Vet. Summary of product characteristics). However, Thomsen, Hansen, and Herskin (2021) found no association between the interval and the calves' immediate behavioral responses. Second, the variation between the studies could be partially attributed to the variation in the amount of procaine deposited around the cornual nerves. Thomsen, Hansen, and Herskin (2021) used a dose of 5 mL procaine (Procamidor Vet, 20 mg mL^−1^) applied bilaterally, which could have resulted in inadequate local anesthesia. On the contrary, the doses in the current study were based on calves' body weights (0.23 mL kg^−1^) and the manufacturer's recommendation. Moreover, the doses were used successfully in a previous study (Adam et al. 2021). Third, the calves were much older in the study of Thomsen, Hansen, and Herskin (2021) (mean age 60.8 days) compared to our current study. The age of the calves might also explain the differences in the efficacy of procaine, as in older calves with well‐developed horn buds, a second injection about 1 cm caudal to the regular cornual nerve block might be required to block the posterior division of the cornual nerve to block sensory input adequately (Clarke, Trim, and Hall 2014). Finally, the variation in the anatomical location of the nerve and associated connective tissue among calves could introduce a level of complexity and potential inconsistency in the efficacy of nerve blocks during disbudding procedures (Venkatachalam et al. 2022). This anatomical variability may impact the accurate targeting of the cornual nerve, potentially influencing the extent and duration of local anesthesia, as recently reviewed by Sheedy et al. (2024). Additionally, the caudolateral part of the horn buds may receive cutaneous innervation from the cornual branch of the second cervical nerve (Valverde and Doherty 2008), needing further blockade as well. Fierheller et al. (2012) reported 87.5% efficacy of cornual nerve block with lidocaine and attributed the failures, among other reasons, to the potential sensory contributions from the second cervical nerve. Further, procaine is metabolized via hydrolysis to para‐aminobenzoic acid (PABA), which inhibits the action of sulfonamides. As such, concurrent treatment with sulfonamides is contraindicated when using procaine (Bishop 2001). Additionally, the PABA metabolite is likely responsible for allergic reactions observed in both humans and animals, particularly when administered intravenously. More studies are necessary to explore these metabolic pathways in calves and assess their clinical implications.

Our results are consistent with previous literature (Heinrich et al. 2010; Mintline et al. 2013; Mirra et al. 2018; Cuttance et al. 2019), suggesting that multimodal analgesia, which includes sedation, local anesthesia, and NSAIDs, can mitigate signs of acute nociception in calves but may not eliminate the development of acute pain and peripheral sensitization. Notably, the local effects of procaine diminished more rapidly than those of procaine‐epinephrine and lidocaine, as evidenced by differences in pain scores and local sensitivity. This difference in duration of efficacy compared to lidocaine can be attributed to procaine's limited protein binding. Local anesthetics vary in their duration of action due primarily to differences in their affinity for protein, where the greater the tendency for protein binding, the longer the anesthetic will sustain neural blockade (Becker and Reed 2012; Taylor and McLeod 2020). Procaine's duration of action is known to be relatively short, ranging from 30 to 60 min (European Medical Agency 1998). Procaine is poorly bound to proteins, including those in the nerve cell membranes, which decreases the duration of the blockade (Tetzlaff 2000) compared to lidocaine. The duration of anesthesia is also influenced by the time a local anesthetic remains near neural fibers (Becker and Reed 2012). Adding epinephrine, a vasoconstrictor, prolongs the effects of procaine up to 45–90 min (European Medicines Agency 1998) by reducing its absorption (Day and Skarda 1991). Moreover, it has been demonstrated that epinephrine can modify certain K^+^ channels in the axons of peripheral nerves through adrenoceptors, thus potentiating the impulse‐blocking action of local anesthetics (Sinnott et al. 2003). Further, local anesthetic nerve block is concentration‐dependent with increased concentrations of local anesthetic, the peak of the action potential is reduced, and impulse conduction is attenuated (Taylor and McLeod 2020). Meanwhile, injecting higher concentrations of the anesthetic agent for blocking peripheral nerves accelerates the onset of block and enhances its duration (Taboada et al. 2006; Fenten et al. 2015) by allowing a greater number of molecules to reach the membrane (Becker and Reed 2012). Hence, when nerves are well‐located or easily accessible, local anesthetic concentration, rather than its dose, is the critical parameter for successful nerve blocks with greater extent and duration (Nakamura et al. 2003).

In contrast, owing to its high liposolubility (partition coefficient of 2.9), lidocaine has a rapid onset of action (0.5–5 min; Fierheller et al. 2012). Moreover, the rate of diffusion across the nerve sheath and nerve membrane is related to the proportion of non‐ionized drug, where local anesthetics with low pKa have a faster onset of action than those with a high pKa (Becker and Reed 2012; Taylor and McLeod 2020). For instance, owing to its low pKa of 7.8, lidocaine has a fast onset because a greater proportion of it exists in the non‐ionized form at a physiologic pH of 7.4 (Taylor and McLeod 2020). In non‐disbudded calves, the anesthetic effect of cornual nerve block with lidocaine lasted 107–512 min (Fierheller et al. 2012), whereas it has been shown to last much shorter (90–180 min) in disbudded ones (Adcock and Tucker 2021). However, the use of lidocaine in food‐producing animals poses a risk to consumers due to potential exposure to its metabolites, such as 2,6‐xylidine, which has genotoxic effects (European Medicines Agency 2015). Further, it has been reported that cornual nerve blocks with lidocaine were averse to calves, most likely due to the acidic pH of the lidocaine solution (Jimenez, Adcock, and Tucker 2019; Adcock and Tucker 2020). Unfortunately, we did not examine this aspect in our study, and no pain was observed during the local blocks' administration with the three local anesthetic products, likely due to the deep sedation of the calves masking any injection pain. Nevertheless, considering the products' pH, it could be assumed that lidocaine (pH 4.94) would be better tolerated by the calves than procaine alone or combined with epinephrine (pH 3.47 and 3.61, respectively). However, more research is needed to establish how calves perceive injections of different local anesthetic solutions.

Changes in plasma cortisol concentrations have been commonly used as an indicator of stress and pain in calves subjected to various painful husbandry procedures, including disbudding (Herskin and Nielsen 2018). However, Stilwell et al. (2010) noted a significantly higher plasma cortisol concentration in xylazine‐sedated calves compared to non‐sedated ones, thus concluded that plasma cortisol should not be used as an indicator of pain in disbudded calves sedated with xylazine. The authors hypothesized that xylazine‐induced analgesia and muscle relaxation may have increased stress in the calves by restricting their ability to respond to human proximity and contact (Stilwell et al. 2010). Likewise, Steckeler et al. (2019) reported a significant increase in plasma cortisol in calves premedicated with im xylazine (0.2 mg kg^−1^) (without any further interventions), lasting for more than 60 min and attributed this to the induced stimulation of α_2_‐adrenoreceptors centrally located in the corticotropin‐releasing hormone neurons by xylazine. Furthermore, α_2_‐adrenoceptor agonists, such as xylazine, are known to initially increase blood pressure due to peripheral vasoconstriction, followed by a decrease in blood pressure due to central sympatholysis, along a reduction in tissue oxygenation in cattle (Hodgson et al. 2002). This physiological strain might also cause rises in cortisol concentrations (Ranheim et al. 2000). Additionally, α_2_‐adrenoceptor agonists can induce nausea across various species by activating receptors in the central chemoreceptor trigger zone (Sinclair 2003). Nausea itself is a stressful physiological response, which activates the hypothalamic–pituitary–adrenal (HPA) axis and leads to increased cortisol release. For instance, in goats, the nausea‐inducing agent cisplatin significantly elevated plasma cortisol levels (Aoyama et al. 2021). The current study observed increased plasma cortisol concentrations before the disbudding. Moreover, there was a negative correlation between behavioral pain scores and cortisol concentrations, indicating that this increment is unlikely to be related to pain. Thus, plasma cortisol may not be sufficient for assessing pain in calves sedated with xylazine, and the results should be interpreted with caution. Therefore, future studies should consider the use of alternative pain biomarkers such as substance P, β‐endorphin, cytokines, and haptoglobin that may provide a more accurate and comprehensive picture of the pain response in calves, especially considering the potential confounding effects of stress‐induced analgesia on cortisol levels.

Study limitations, methodological considerations, and future prospects: First, the study lacked a handling‐only control group, which would have allowed for a clearer comparison of cortisol levels in response to handling stress alone versus disbudding‐related pain. Although our results provide meaningful comparisons between different anesthetic protocols, the absence of a control group limits the ability to fully isolate the effects of disbudding from the handling procedure. Additionally, we did not include a lidocaine‐epinephrine group for comparison, which would have allowed for a direct evaluation of the effects of epinephrine in combination with lidocaine, as was done with procaine. This comparison is crucial for fully evaluating alternative anesthetic protocols, and future studies should address this gap to offer a comprehensive evaluation of alternative anesthetic protocols for disbudding. Second, to maintain consistency with established disbudding protocols, xylazine was a necessary choice for sedation. However, we recognize the concerns regarding the accumulation of its metabolite, 2,6‐xylidine, which has been associated with potential genotoxic effects. The European Medicines Agency has reviewed these concerns and concluded that, while there is a risk for genotoxic effects with exposure to this metabolite, it has not been detected in cattle milk or tissues. As a result, it was determined that there is no significant consumer safety concern regarding xylazine in cattle (European Medicines Agency 2015). Nonetheless, future research should focus on identifying alternative sedative protocols that can avoid the production of 2,6‐xylidine. Third, an important methodological consideration is the assessment of local anesthesia efficacy. While all calves were evaluated for skin sensitivity using a needle prick test prior to disbudding, few still exhibited reactions during the disbudding procedure. This suggests that the absence of response to needle prick may overestimate the degree of the nerve block achieved, potentially leaving some calves inadequately anesthetized during disbudding. Therefore, a different or more sensitive method for assessing the completeness of local anesthesia during thermocautery disbudding should be developed to ensure reliable and complete nerve blocks for such procedures. Lastly, another limitation lies in the reproducibility of the methods, as the study employed a single observer to assess pain responses and efficacy of anesthesia. The lack of multiple observers reduces the ability to assess inter‐observer variability. Additionally, although the observer was blinded, the assistants restraining the calves were not, which may have inadvertently introduced bias into the assessments. To improve the robustness and reproducibility of future studies, it would be important to implement complete blinding of all personnel involved in the disbudding procedure and data collection. By addressing these limitations in future research, a more comprehensive and accurate evaluation of pain management strategies for disbudding in calves can be achieved.

In conclusion, our study suggests that epinephrine prolongs the action of procaine, and the duration of the anesthetic effect of procaine‐epinephrine is comparable to lidocaine in connection to disbudding. Consequently, procaine‐epinephrine appears to be an efficient and safe alternative to lidocaine for cornual nerve block in calves before thermocautery disbudding. However, further large‐scale investigations to confirm these findings, possibly exploring higher doses and examining the efficacy of lidocaine combined with epinephrine for prolonged pain alleviation after disbudding, are warranted. Cortisol concentrations as a pain indicator in disbudded calves sedated with xylazine are questioned.

## Author Contributions

**Magdy Adam:** study design, data acquisition, data analysis, drafting manuscript. **Kati Salla:** study design, data acquisition, critical review and editing of the manuscript. **Marja Raekallio, Annemari Jokela, and Riikka Aho:** data acquisition, critical review and editing of the manuscript. **Laura Hänninen:** study design, critical review and editing of the manuscript. **Ann‐Helena Hokkanen:** study design, funding acquisition, data analysis, drafting manuscript. All authors have read and approved the manuscript.

## Ethics Statement

The authors confirm that the ethical policies of the journal, as noted on the journal's author guidelines page, have been adhered to and the appropriate ethical review committee approval has been received. The authors confirm that they have adhered to European standards for the protection of animals used for scientific purposes.

## Conflicts of Interest

The authors declare no conflicts of interest.

## Data Availability

The data that support the findings of this study are available from the corresponding author upon reasonable request.

## Appendix A

Pain Score Adapted From Mirra et al. (2018).

**Table:**

General subjective assessment	0 = no signs of pain
	1 = mild signs of pain
	2 = moderate signs of pain
	3 = severe signs of pain
	4 = unbearable pain
Interactive behavior (1 min)	0 = interest
	1 = some interest
	2 = no interest
Postural behavior	1 = head low, asymmetric ears
	1 = corrugated eyes
	1 = reduced grooming
	1 = restlessness
	1 = apathy
	1 = abnormal position
	1 = other
Peri‐bud palpation	0 = no reaction
	1 = mild reaction
	2 = moderate reaction
	3 = severe reaction
Foot stamping (1 min)	0 = no
	1 = yes
Tail flicking (1 min)	0 = no
	1 = yes
Ear flicking (1 min)	0 = no
	1 = yes
Head rubbing (1 min)	0 = no
	1 = nes
Head shaking (1 min)	0 = yo
	1 = nes
	Total (0–21)

## Appendix B

Reaction to Von Frey Filaments Score.

**Table:**

g mm^−2^	Score
100	0
26, 60	1
10, 15	2
4, 6, 8	3
1, 1.4, 2	4
0.16, 0.4, 0.6	5
0.02, 0.04, 0.07	6

## References

[jvp13493-bib-0001] Adam, M. , K. Salla , R. Aho , et al. 2021. “A Comparison of Sedative Effects of Xylazine Alone or Combined With Levomethadone or Ketamine in Calves Prior to Disbudding.” Veterinary Anaesthesia and Analgesia 48: 906–913. 34602358 10.1016/j.vaa.2021.08.004

[jvp13493-bib-0002] Adcock, S. J. J. , D. M. Cruz , and C. B. Tucker . 2020. “Behavioral Changes in Calves 11 Days After Cautery Disbudding: Effect of Local Anesthesia.” Journal of Dairy Science 103: 8518–8525. 32564957 10.3168/jds.2020-18337

[jvp13493-bib-0003] Adcock, S. J. J. , B. C. Downey , C. Owens , and C. B. Tucker . 2023. “Behavioral Changes in the First 3 Weeks After Disbudding in Dairy Calves.” Journal of Dairy Science 106: 6365–6374. 10.3168/jds.2023-23237. 37500438

[jvp13493-bib-0004] Adcock, S. J. J. , and C. B. Tucker . 2020. “Conditioned Place Preference Reveals Ongoing Pain in Calves 3 Weeks After Disbudding.” Scientific Reports 10: 3849. 32123190 10.1038/s41598-020-60260-7 PMC7052132

[jvp13493-bib-0005] Adcock, S. J. J. , and C. B. Tucker . 2021. “Injury Alters Motivational Trade‐Offs in Calves During the Healing Period.” Scientific Reports 11: 6888. 10.1038/s41598-021-86313-z. 33767288 PMC7994642

[jvp13493-bib-0006] Aoyama, M. , M. Shioya , Y. Tsukamoto , H. Hasegawa , and S. Sugita . 2021. “The Effects of Cisplatin, an Emetic Agent, on Behavior and Plasma Cortisol Levels in Goats.” Animal Science Journal 92: e13607. 34318567 10.1111/asj.13607 PMC9286031

[jvp13493-bib-0007] Bates, A. J. , R. A. Laven , F. Chapple , and D. S. Weeks . 2016. “The Effect of Different Combinations of Local Anaesthesia, Sedative and Non‐steroidal Anti‐Inflammatory Drugs on Daily Growth Rates of Dairy Calves After Disbudding.” New Zealand Veterinary Journal 64: 282–287. 27256490 10.1080/00480169.2016.1196626

[jvp13493-bib-0008] Bates, A. J. , M. A. Sutherland , F. Chapple , et al. 2019. “A New Method of Administering Local Anesthesia for Calf Disbudding: Findings From a Comparative on‐Farm Study in New Zealand.” Journal of Dairy Science 102: 2492–2506. 30638993 10.3168/jds.2018-15033

[jvp13493-bib-0009] Becker, D. E. , and K. L. Reed . 2012. “Local Anesthetics: Review of Pharmacological Considerations.” Anesthesia Progress 59: 90–101. 22822998 10.2344/0003-3006-59.2.90 PMC3403589

[jvp13493-bib-0010] Bishop, Y. 2001. The Veterinary Formulary. 5th ed. Cambridge: Pharmaceutical Press.

[jvp13493-bib-0011] Casoni, D. , A. Mirra , M. R. Suter , A. Gutzwiller , and C. Spadavecchia . 2019. “Can Disbudding of Calves (One Versus Four Weeks of Age) Induce Chronic Pain?” Physiology & Behavior 199: 47–55. 30414886 10.1016/j.physbeh.2018.11.010

[jvp13493-bib-0012] Clarke, K. W. , C. M. Trim , and L. W. Hall . 2014. “Anaesthesia of Cattle.” In Veterinary Anaesthesia, 11th ed., 313–343. Philadelphia, PA: W.B. Saunders.

[jvp13493-bib-0013] Council Directive No 2008/119/EC of 18 December 2008 . 2008. “Laying Down Minimum Standards for the Protection of Calves.” Official Journal of the European Union L 10: 7–13. Accessed February 12, 2024. https://eur‐lex.europa.eu/eli/dir/2008/119/oj.

[jvp13493-bib-0014] Council Regulation (EC) No 2019/6 of the European Parliament and of the Council of 11 December 2018 on Veterinary Medicinal Products and Repealing Directive 2001/82/EC . 2019. Official Journal of the European Union. Accessed January 29, 2024. https://eur‐lex.europa.eu/eli/reg/2019/6/oj.

[jvp13493-bib-0015] Cozzi, G. , F. Gottardo , M. Brscic , et al. 2015. “Dehorning of Cattle in the EU Member States: A Quantitative Survey of the Current Practices.” Livestock Science 179: 4–11.

[jvp13493-bib-0016] Cuttance, E. L. , W. A. Mason , D. A. Yang , R. A. Laven , J. McDermott , and K. Inglis . 2019. “Effects of a Topically Applied Anaesthetic on the Behaviour, Pain Sensitivity and Weight Gain of Dairy Calves Following Thermocautery Disbudding With a Local Anaesthetic.” New Zealand Veterinary Journal 67: 295–305. 31272290 10.1080/00480169.2019.1640651

[jvp13493-bib-0017] Day, T. K. , and R. T. Skarda . 1991. “The Pharmacology of Local Anesthetics.” Veterinary Clinics of North America. Equine Practice 7: 489–500. 1820222 10.1016/s0749-0739(17)30482-0

[jvp13493-bib-0018] European Medicines Agency . 1998. “EMA/MRL/217/97‐FINAL/1998—Committee for Veterinary Medicinal Products, Procaine: Summary Report.” https://www.ema.europa.eu/en/documents/mrl‐report/procaine‐summary‐report‐committee‐veterinary‐medicinal‐products_en.pdf.

[jvp13493-bib-0019] European Medicines Agency . 2015. “EMA/CVMP/118717/2015—CVMP Assessment report regarding the request for an opinion under Article 30(3) of Regulation (EC) No 726/2004: In relation to the potential risk for the consumer resulting from the use of lidocaine in food producing species.” Accessed June 13, 2024. https://www.ema.europa.eu/en/documents/report/cvmp‐assessment‐report‐regarding‐request‐opinion‐under‐article‐303‐regulation‐ec‐no‐7262004‐relation‐potential‐risk‐consumer‐resulting‐use‐lidocaine‐food‐producing‐spec_en.pdf.

[jvp13493-bib-0020] European Medicines Agency . 2019. “Procamidor Duo 40 mg/ml + 0.036 mg/ml Solution for Injection for Animals.” Accessed October 28, 2024. https://medicines.health.europa.eu/veterinary/en/600000014952.

[jvp13493-bib-0021] Fenten, M. G. , K. P. Schoenmakers , P. J. Heesterbeek , G. J. Scheffer , and R. Stienstra . 2015. “Effect of Local Anesthetic Concentration, Dose and Volume on the Duration of Single‐Injection Ultrasound‐Guided Axillary Brachial Plexus Block With Mepivacaine: A Randomized Controlled Trial.” BMC Anesthesiology 30, no. 15: 130. 10.1186/s12871-015-0110-0 PMC4588252 26423050

[jvp13493-bib-0022] Fierheller, E. E. , N. A. Caulkett , D. B. Haley , D. Florence , and L. Doepel . 2012. “Onset, Duration and Efficacy of Four Methods of Local Anesthesia of the Horn Bud in Calves.” Veterinary Anaesthesia and Analgesia 39: 431–435. 22524418 10.1111/j.1467-2995.2012.00717.x

[jvp13493-bib-0023] Heinrich, A. , T. F. Duffield , K. D. Lissemore , and S. T. Millman . 2010. “The Effect of Meloxicam on Behavior and Pain Sensitivity of Dairy Calves Following Cautery Dehorning With a Local Anesthetic.” Journal of Dairy Science 93: 2450–2457. 20494153 10.3168/jds.2009-2813

[jvp13493-bib-0024] Herskin, M. S. , and B. H. Nielsen . 2018. “Welfare Effects of the Use of a Combination of Local Anesthesia and NSAID for Disbudding Analgesia in Dairy Calves‐Reviewed Across Different Welfare Concerns.” Frontiers in Veterinary Science 5: 117. 29922684 10.3389/fvets.2018.00117 PMC5996095

[jvp13493-bib-0025] Hodgson, D. S. , C. I. Dunlop , P. L. Chapman , and J. A. Smith . 2002. “Cardiopulmonary Effects of Xylazine and Acepromazine in Pregnant Cows in Late Gestation.” American Journal of Veterinary Research 63: 1695–1699. 12492284 10.2460/ajvr.2002.63.1695

[jvp13493-bib-0026] Huber, J. , T. Arnholdt , E. Möstl , C. C. Gelfert , and M. Drillich . 2013. “Pain Management With Flunixin Meglumine at Dehorning of Calves.” Journal of Dairy Science 96: 132–140. 10.3168/jds.2012-5483. 23182358

[jvp13493-bib-0027] Jimenez, R. E. , S. J. J. Adcock , and C. B. Tucker . 2019. “Acute Pain Responses in Dairy Calves Undergoing Cornual Nerve Blocks With or Without Topical Anesthetic.” Journal of Dairy Science 102: 3431–3438. 30772020 10.3168/jds.2018-15445

[jvp13493-bib-0028] Mintline, E. M. , M. Stewart , A. R. Rogers , et al. 2013. “Play Behavior as an Indicator of Animal Welfare: Disbudding in Dairy Calves.” Applied Animal Behaviour Science 144: 22–30.

[jvp13493-bib-0029] Mirra, A. , C. Spadavecchia , R. Bruckmaier , A. Gutzwiller , and D. Casoni . 2018. “Acute Pain and Peripheral Sensitization Following Cautery Disbudding in 1‐ and 4‐Week‐Old Calves.” Physiology & Behavior 184: 248–260. 29221809 10.1016/j.physbeh.2017.11.031

[jvp13493-bib-0030] Nakamura, T. , F. Popitz‐Bergez , J. Birknes , and G. R. Strichartz . 2003. “The Critical Role of Concentration for Lidocaine Block of Peripheral Nerve In Vivo: Studies of Function and Drug Uptake in the Rat.” Anesthesiology 99: 1189–1197. 14576558 10.1097/00000542-200311000-00028

[jvp13493-bib-0031] Nielsen, S. S. , J. Alvarez , D. J. Bicout , et al. 2023. “Welfare of Calves.” EFSA Journal 21: e07896. 37009444 10.2903/j.efsa.2023.7896 PMC10050971

[jvp13493-bib-0032] Ranheim, B. , T. E. Horsberg , N. E. Søli , K. A. Ryeng , and J. M. Arnemo . 2000. “The Effects of Medetomidine and Its Reversal With Atipamezole on Plasma Glucose, Cortisol and Noradrenaline in Cattle and Sheep.” Journal of Veterinary Pharmacology and Therapeutics 23: 379–387. 11168916 10.1046/j.1365-2885.2000.00291.x

[jvp13493-bib-0033] Richter Pharma, A. G. 2017. “Procamidor Vet. Summary of Product Characteristics.” Accessed November 20, 2023. https://www.richterpharma.com/fileadmin/media/SPC/SPC_Procamidor_ENG_2017.pdf.

[jvp13493-bib-0034] Sheedy, D. B. , S. S. Aly , C. B. Tucker , and T. W. Lehenbauer . 2024. “Mini‐Review: The History and Future of the Cornual Nerve Block for Calf Disbudding.” JDS Communications 5: 305–309. 10.3168/jdsc.2023-0506. 39220846 PMC11365352

[jvp13493-bib-0035] Sinclair, M. D. 2003. “A Review of the Physiological Effects of Alpha2‐Agonists Related to the Clinical Use of Medetomidine in Small Animal Practice.” Canadian Veterinary Journal 44: 885–897. PMC385445 14664351

[jvp13493-bib-0036] Sinnott, C. J. , L. P. Cogswell III , A. Johnson , and G. R. Strichartz . 2003. “On the Mechanism by Which Epinephrine Potentiates Lidocaine's Peripheral Nerve Block.” Anesthesiology 98: 181–188. 12502995 10.1097/00000542-200301000-00028

[jvp13493-bib-0037] Steckeler, P. , D. Fux , M. Metzner , G. Knubben , A. Rieger , and C. Baumgartner . 2019. “The Course of Plasma Cortisol Concentration After Three Different Doses of Ketamine in Xylazine‐Premedicated Calves.” Veterinary Anaesthesia and Analgesia 46: 335–343. 30956016 10.1016/j.vaa.2018.11.005

[jvp13493-bib-0038] Stilwell, G. , R. C. Carvalho , N. Carolino , M. S. Lima , and D. M. Broom . 2010. “Effect of Hot‐Iron Disbudding on Behaviour and Plasma Cortisol of Calves Sedated With Xylazine.” Research in Veterinary Science 88: 188–193. 19647841 10.1016/j.rvsc.2009.06.012

[jvp13493-bib-0039] Taboada, M. , J. Rodríguez , C. Valiño , et al. 2006. “What Is the Minimum Effective Volume of Local Anesthetic Required for Sciatic Nerve Blockade? A Prospective, Randomized Comparison Between a Popliteal and a Subgluteal Approach.” Anesthesia and Analgesia 102: 593–597. 16428568 10.1213/01.ane.0000189188.08679.2a

[jvp13493-bib-0040] Taylor, A. , and G. McLeod . 2020. “Basic Pharmacology of Local Anaesthetics.” BJA Education 20: 34–41.33456928

[jvp13493-bib-0041] Tetzlaff, J. E. 2000. “The Pharmacology of Local Anesthetics.” Anesthesiology Clinics of North America 18: 217–233. 10935008 10.1016/s0889-8537(05)70161-9

[jvp13493-bib-0042] Thomsen, P. T. , J. H. Hansen , and M. S. Herskin . 2021. “Dairy Calves Show Behavioural Responses to Hot Iron Disbudding After Local Anaesthesia With Procaine.” Veterinary Record 188: e52. 34651735 10.1002/vetr.52

[jvp13493-bib-0043] Valverde, A. , and T. J. Doherty . 2008. “Anesthesia and Analgesia of Ruminants.” In Anesthesia and Analgesia in Laboratory Animals, 2nd ed., 345–411. Cambridge, MA: Academic Press.

[jvp13493-bib-0044] Venkatachalam, D. , N. Kells , P. Chambers , A. Jacob , N. Ward , and P. Singh . 2022. “Pharmacokinetics and Efficacy of a Novel Long‐Acting Bupivacaine Formulation for Cornual Nerve Block in Calves.” Frontiers in Veterinary Science 9: 1060951. 36532336 10.3389/fvets.2022.1060951 PMC9751437

[jvp13493-bib-0045] Winder, C. B. , C. L. Miltenburg , J. M. Sargeant , et al. 2018. “Effects of Local Anesthetic or Systemic Analgesia on Pain Associated With Cautery Disbudding in Calves: A Systematic Review and Meta‐Analysis.” Journal of Dairy Science 101: 5411–5427. 10.3168/jds.2017-14092. 29550129

